# Serum HER2 Level Measured by Dot Blot: A Valid and Inexpensive Assay for Monitoring Breast Cancer Progression

**DOI:** 10.1371/journal.pone.0018764

**Published:** 2011-04-13

**Authors:** Li-Duan Tan, Yuan-Yuan Xu, Yue Yu, Xiao-Qing Li, Ying Chen, Yu-Mei Feng

**Affiliations:** 1 Department of Biochemistry and Molecular Biology, Tianjin Medical University Cancer Institute and Hospital, Tianjin, China; 2 Key Laboratory of Breast Cancer Prevention and Treatment of the Ministry of Education, Tianjin Medical University Cancer Institute and Hospital, Tianjin, China; 3 Clinical Laboratory, Tianjin Medical University Cancer Institute and Hospital, Tianjin, China; Enzo Life Biosciences, United States of America

## Abstract

Human epidermal growth factor receptor 2 (HER2) is one of the most important prognostic and predictive factors for breast cancer patients. Recently, serum HER2 ECD level of patients detected by enzyme-linked immunoabsorbent assay (ELISA) has been shown to predict tumor HER2 status and reveal its association with tumor progression, recurrence and poor prognosis. In this study, we established a new method, dot blot assay, to measure the serum HER2 level in breast cancer patients and further to evaluate the clinical value for monitoring breast cancer progression. We found that the serum HER2 level measured by dot blot assay was significantly correlated with tissue HER2 status in breast cancer patients (*P* = 0.001), and also significantly correlated with HER2 level measured by ELISA (*P* = 1.06×10^−11^). Compared with ELISA method, the specificity and sensitivity of dot blot assay were 95.3% and 65.0%, respectively. The serum HER2 levels of patients with grade III or ER-negative were higher than those with grade I–II (*P* = 0.004) and ER-positive (*P* = 0.033), respectively. Therefore, the novel dot blot method to detect serum HER2 level is a valid and inexpensive assay with potential application in monitoring breast cancer progression in clinical situations.

## Introduction

Breast cancer is one of most common malignant tumors with the highest incidence and the second highest mortality among all malignant tumors in females [1]. Molecular techniques have greatly promoted the detection and prediction of prognosis and treatment response of breast cancer. An important instance is the detection of overexpression of human epidermal growth factor receptor 2 (HER2), which is present in 10–25% of breast cancer [2]. HER2 is a transmembrane tyrosine kinase receptor belonging to a family of epidermal growth factor receptors structurally related to the epidermal growth factor receptor (EGFR), and is encoded by *ERBB2/HER2* oncogene located on chromosome 17q21 [3], [4]. Deregulated expression of HER2 has been implicated in the development of numerous types of human cancers [5]. Amplification of *ERBB2* gene or overexpression of HER2 protein is one of the most important prognostic factors in breast cancer patients [3], [6], [7] Therefore, HER2 has been a target for specific breast cancer treatment with the monoclonal antibody trastuzumab (Herceptin®) which was approved in 1998 by the United States Food and Drug Administration (FDA). The benefit of this particular therapy has been sufficiently confirmed in metastatic breast carcinomas [8]. In addition to trastuzumab, other therapeutic strategies have been developed recently to target the HER2 protein, such as the tyrosine kinase inhibitor lapatinib, which appears to have efficacy after failure of trastuzumab therapy [9].

HER2 status of breast cancer is routinely assessed by either immunohistochemical (IHC) analysis of HER2 protein or by fluorescent in situ hybridization (FISH) analysis of *ERBB2* gene copy number in primary tumor tissues. Recently it was shown that HER2 extracellular domain (ECD) can be shed into the circulation by proteolytic cleavage from the full-length HER2 receptor, and is detected in the serum of women with benign breast disease, primary and metastatic breast cancer [10]. Overwhelming evidence demonstrated that serum HER2 level has the potential value to predict tumor HER2 status as detected by IHC [11], [12] and is associated with tumor progression, recurrence and poor prognosis [13], [14], [15]. In fact, assay for serum HER2 ECD level has been approved by the FDA for the follow-up and monitoring of patients undergoing various treatments for metastatic breast cancer [11], [16]. Up to now, the “soluble” receptor is mainly analyzed with the enzyme-linked immunoabsorbent assay (ELISA) method [15]. In the present study, we established a new method, dot blot, to measure the serum HER2 level and further evaluated their clinical value for predicting tumor HER2 status and tumor progression.

## Results

### HER2 expression in primary breast cancer tissues

**Figure 1:**
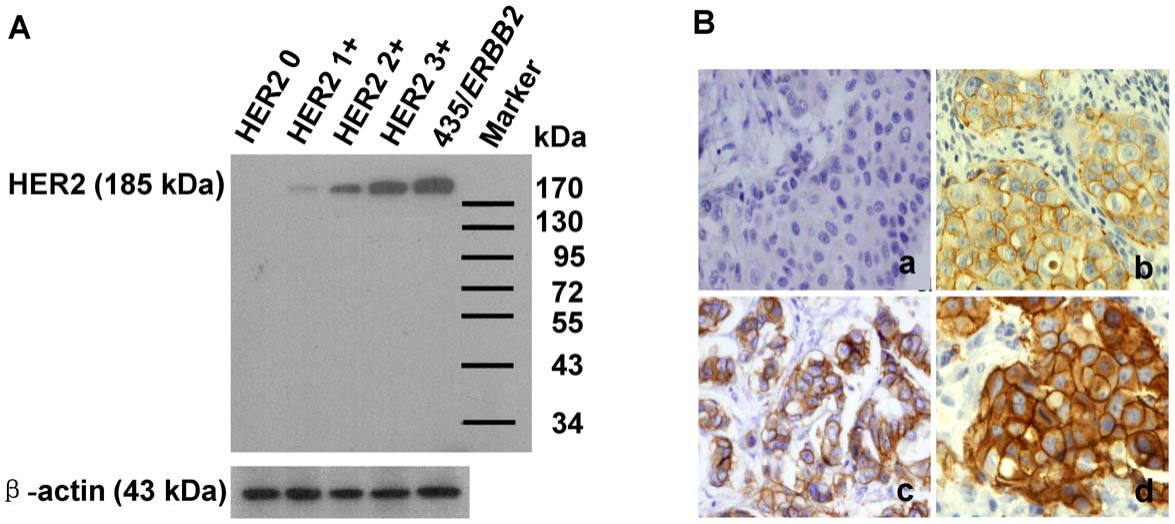
HER2 expression in primary breast cancer tissues. (**A**), Western blot analysis of HER2 in representative primary breast cancer tissues and MDA-MB-435/*ERBB2* cell line (top panel). β-actin served as loading control (bottom panel). (**B**), Representative IHC staining for HER2 in breast cancer tissues classified as negative (0; a), weak positive (1+; b), moderate positive (2+; c), and strong positive (3+; d) for HER2 expression.

The western blot showed that the HER2 antibody is very specific, detecting the p185 HER2 protein (Fig. 1A). Thus this antibody is suitable to be used in IHC staining to detect tissue HER2 status and dot blot assay to detect the serum HER2 level. Representative HER2 immunostaining of primary breast cancer tissues is shown in Fig. 1B and the localization of HER2 was restricted to the cell membrane. Among 126 cases, 64.3% (81/126) patients were HER2 negative (0), 13.5% (17/126) patients were weakly positive (1+), 15.1% (19/126) were moderately positive (2+), and 7.1% (9/126) were strongly positive (3+).

### Serum HER2 levels was detected by dot blot

**Figure 2:**
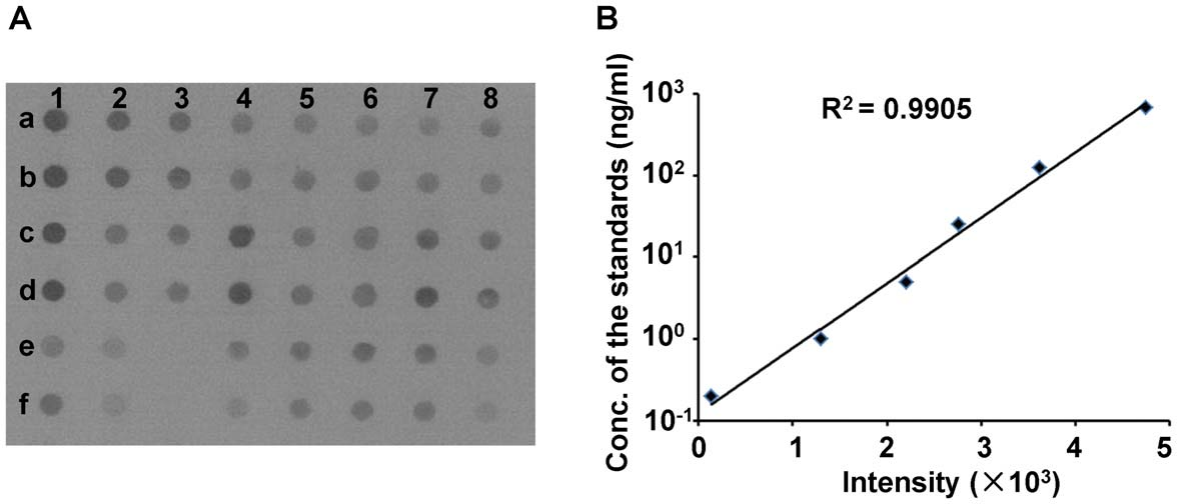
The serum HER2 level detected by dot blot assay. (**A**), The digitized image of the dot blot assay of the soluble serum HER2. Samples a1 to a7 are standards, sample a8 is the blank control, rows c and e are the tested samples, and rows b, d, and f are duplicates of a, c, e, respectively. (**B**), The standard curve of soluble HER2 levels.

**Figure 3:**
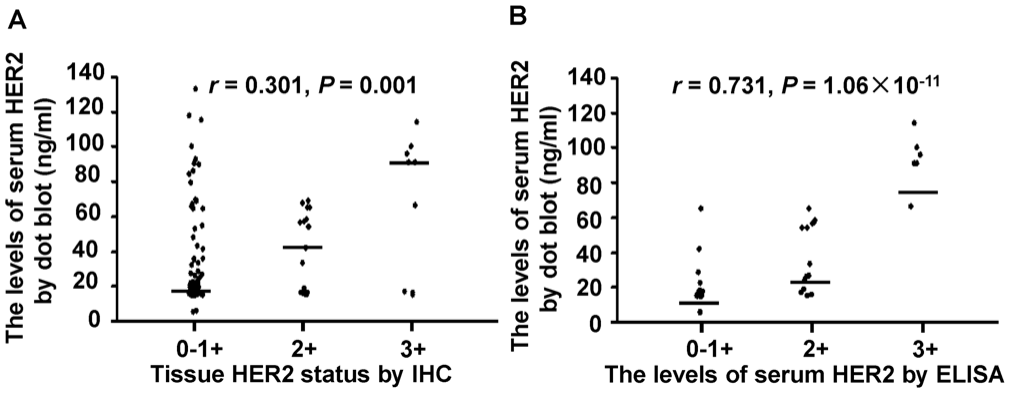
Correlations among HER2 status detected by IHC, ELISA and Dot blot assay. (**A**), Correlation between tissue HER2 status and serum HER2 levels. The horizontal dash lines indicated the median values in each group. (**B**), Correlation between dot blot assay and ELISA for the detection of serum HER2 levels. The horizontal dash lines indicate the median values in each group.

By dot blot assay, the concentration of serum HER2 in 133 breast cancer patients were determined to range from 0.7 ng/ml to 133.1 ng/ml, median 20.5 ng/ml (Fig. 2). All samples were divided into four groups according to the baseline serum HER2 levels: negative HER2 (0; less than 19 ng/ml), weakly positive HER2 (1+; from 19 to 32 ng/ml), moderately positive HER2 (2+; from 32 to 66 ng/ml), and strongly positive HER2 (3+; more than 66 ng/ml). Notably, the serum HER2 levels detected by dot blot assay and tissue HER2 status examined by IHC were positively correlated in 126 primary breast cancer patients (Spearman's rho = 0.301, *P* = 0.001; Table 1, Fig. 3A).

**Table 1:** Correlations among HER2 status detected by IHC, ELISA and Dot blot assay.

HER2 status	Cases	Median serum HER2 levels by dot blot (ng/ml)	Rank sum tests		Serum HER2 levels by dot blot			Spearman rank correlation	
			**Z**/χ^2^	***P***	**0–1+**	**2+**	**3+**	***r***	***P***
IHC for tissue	126								
0–1+	98	18.0	7.291	0.026	72 (73.4)	13 (13.3)	13 (13.3)	0.301	0.001
2+	19	42.1			8 (42.1)	9 (47.4)	2 (10.5)		
3+	9	90.9			3 (33.3)	0 (0.0)	6 (66.7)		
ELISA for serum									
0–1+	43	15.9	29.058	4.90×10^−12^	41 (95.3)	2 (4.7)	0 (0.0)	0.731	1.06×10^−11^
2+	14	30.5			7 (50.0)	7 (50.0)	0 (0.0)		
3+	6	93.7			0 (0.0)	0 (0.0)	6 (100.0)		

### Correlation between ELISA and dot blot in serum HER2 detection

A total of 63 patients showed correlation between serum HER2 levels detected by dot blot and tissue HER2 status detected by IHC. The serum HER2 levels of these 63 patients were measured using ELISA and found to be in the range of 2.3 to 112.5 ng/ml. Among the patients, 18 (28.6%) were judged as HER2 (0; less than 15 ng/ml), 25 (39.7%) were HER2 (1+; from 15 to 28 ng/ml), 14 (22.2%) were HER2 (2+; from 28 to 59 ng/ml), and 6 (9.5%) were HER2 (3+; more than 59 ng/ml). Most significantly, the serum HER2 levels detected by ELISA and dot blot assay were positively correlated (Spearman's rho = 0.731, *P* = 1.06×10^−11^; Table 1, Fig. 3B). The HER2 0/1+ was grouped into the low expression group, which should not receive trastuzumab treatment. Compared with the ELISA, the specificity (HER2; 0/1+) and sensitivity (HER2; 2+/3+) of the dot blot assay were 95.3% (41/43) and 65.0% (13/20), respectively.

### Relationship between baseline serum HER2 levels and clinicopathological variables

By dot blot assay, the serum HER2 levels of patients with grade III or ER-negative were higher than those with grade I–II (*P* = 0.004) or ER-positive (*P* = 0.033), respectively. No significant relationships were found between HER2 levels and other clinicopathological variables, such as age, menopausal status, clinical stage, lymph nodes involvement or PR status. Moreover, we compared serum HER2 level in invasive and non-invasive ductal carcinoma and found that serum HER2 levels were higher in patients with invasive ductal carcinoma than patients with ductal carcinoma in situ, but no significant difference was revealed between these two groups since there were only 8 cases of ductal carcinoma in situ (Table 2).

**Table 2:** Association between serum HER2 levels and clinicopathological variables (N = 133).

Variables	Cases	Serum HER2 levels by dot blot			Chi-squae test	
		0–1+(%)	2+ (%)	3+ (%)	χ^2^	*P*
Age (years)						
≤45	33	20 (60.6)	10 (30.3)	3 (9.1)	6.394	0.172
45–55	47	31 (66.0)	7 (14.9)	9 (19.1)		
>55	53	36 (67.9)	6 (11.3)	11 (20.8)		
Menopausal status						
Premenopausal	60	40 (66.7)	14 (23.3)	6 (10.0)	5.695	0.058
Postmenopausal	73	47 (64.4)	9 (12.3)	17 (23.3)		
Pathology diagnosis						
Ductal carcinoma in situ	8	6 (75.0)	0 (0.0)	2 (25.0)	0.884	0.390
Invasive ductal carcinoma	125	81 (64.8)	23 (18.4)	21 (16.8)		
Tumor size (cm)						
≤2	44	31 (70.5)	9 (20.5)	4 (9.0)	3.042	0.218
>2	71	48 (67.6)	9 (12.7)	14 (19.7)		
Missing	18					
Clinical stage						
I	40	30 (75.0)	4 (10.0)	6 (15.0)	1.497	0.473
II+III	84	55 (65.5)	15 (17.9)	14 (16.6)		
Missing	9					
Histological grade						
I+II	86	62 (72.1)	15 (17.4)	9 (10.5)	10.906	0.004
III	14	5 (35.7)	3 (21.4)	6 (42.9)		
Missing	33					
Lymph node status						
Negative	75	51 (68.0)	9 (12.0)	15 (20.0)	5.510	0.064
Positive	48	30 (62.5)	13 (27.1)	5 (10.4)		
Missing	10					
ER status						
Positive	71	49 (69.0)	15 (21.1)	7 (9.9)	6.844	0.033
Negative	53	33 (62.3)	6 (11.3)	14 (26.4)		
Missing	9					
PR status						
Positive	40	30 (75.0)	7 (17.5)	3 (7.5)	3.818	0.148
Negative	84	52 (61.9)	14 (16.7)	18 (21.4)		
Missing	9					

## Discussion

Amplification of *ERBB2* oncogene and/or overexpression of HER2 protein in breast cancer have been linked to poor prognosis and a differential response to a variety of systemic treatments [11], therefore, HER2 status plays an important role in the prognosis and prediction for breast cancer patients. With the availability of the monoclonal antibody trastuzumab as an effective therapy for metastatic breast cancer, there is an increased need to evaluate HER2 status to identify those patients who might benefit from this treatment and to monitor disease progression. Clinically, HER2 status is most often determined in tissues from primary or metastatic breast cancer by IHC assay that examines the protein expression or by FISH assay that determines gene amplification. Results of these tests are generally in agreement, although discrepancies are possible due to inherent variability in procedures, lack of standardization and subjective interpretation [17].

Interestingly, several lines of recent evidence suggest that HER2 serum level might provide a novel and useful tool for management of patients with breast cancer, as it might predict prognosis, treatment selection and clinical response. Isola et al [18] demonstrated the utility of serum HER2 for monitoring the tumor progression of patients with HER2-positive breast carcinoma. Kandl et al [19] reported a correlation between elevated serum HER2 and poor prognosis of advanced breast carcinoma. In addition, both Willsher et al [20] and Rocca et al [21] showed the prognostic importance of serum HER2 in early breast carcinoma patients. Molina et al [22] demonstrated that abnormal presurgical serum HER2 was related to the poor prognosis of node-positive and node-negative breast carcinoma. Furthermore, Ludovini et al [23] showed a shorter disease-free survival in patients with elevated serum HER2 ECD levels. Most notably, assay for serum HER2 ECD has been approved by the FDA for the follow-up and monitoring of patients undergoing treatment for metastatic breast cancer on various therapies [11], [16], [24]. Therefore, the reassessment of HER2 status based on HER2 serum level during disease progression might help optimize treatment strategy by identifying patients who could profit from trastuzumab or other HER2 targeted therapy [25], [26] There is good evidence showing that shedding of HER2 ECD only occurs in those patients whose tumors show HER2 amplification/overexpression [27]. In our present study, we established a novel method, dot blot, for the detection of serum HER2 levels, and found statistically significant correlation between the tissue HER2 status as detected by IHC and the serum HER2 level as detected by dot blot assay. Moreover, serum HER2 level determined by dot blot assay showed a significant correlation with that determined by ELISA, the most widely used method to detect the serum HER2 ECD level. Strikingly, the results of dot blot assay were more consistent with IHC analysis. Thus our results suggested that serum HER2 levels determined by dot blot assay might aid the assessment of HER2 status since this method is much less subjected to the investigator's subjective view.

Based on a systematic review of the literature, Carney et al [28] reported that the prevalence of elevated serum HER2 ECD levels was approximately 18% (range 0–38%) in primary breast cancer (2,623 women in 25 studies) but increased to 43% (range 23–80%) in metastatic breast cancer (4,622 women in 45 studies). However, our results indicated that the elevated serum HER2 levels were 25.0% (2+/3+) in non-invasive ductal carcinoma and 35.2% (2+/3+) in invasive ductal carcinoma. The discrepancy may be caused by the specificity of HER2 antibody, small sample size, different standards for positive, different histological types of breast cancer and the ethnic differences. Consistent with the report by Ludovini et al [23], the elevated serum HER2 level was observed in patients with high histological grade and ER negativity, suggesting that these patients had a high risk of metastasis. Taken together, in this study, serum HER2 level determined by dot blot can serve as a biomarker for the prediction of aggressive tumor and progression of breast cancer.

Compared with ELISA, which requires the inclusion of pre-coating antibody, dot blot assay greatly reduces the amount of antibody and the sample. In this study, we arrayed serum samples on solid phase membrane to concentrate protein. For the 48-point density, only 2 µl serum of each sample is needed. In addition, only 5 µg antibody per 96-well is required, much less than 20 µg antibody per 96-well required for ELISA. Therefore, the cost of dot blot is much cheaper than ELISA. Based on this method, increasing the dot density to make a high-density array will significantly reduce the amount of serum samples and the antibody required for each sample. This method is especially suitable for testing a large number of samples. Further optimization of this dot blot assay is necessary in terms of the sample print system, detection system and analysis system.

Because of the difficulties associated with standardization of preanalytical and analytical as well as postanalytical factors, the use of experimental controls is essential to guarantee reliable results. Each array should include negative (normal serum) and positive (known concentration of serum HER2) controls. The mean (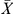) and standard deviation (s) of quality controls are calculated by multiple batches. If the concentration of quality controls is within 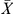
±2s, the assay is considered reliable results.

In conclusion, to our knowledge, this is the first report of the development and evaluation of dot blot assay for the detection of serum HER2 level in breast cancer patients. Although we demonstrate that this assay showed significant correlation with ELISA as well as IHC, due to the limited size of patients, our results need to be confirmed in large prospective trials before the simple and inexpensive dot blot assay can be routinely used in the clinical for monitoring breast cancer progression.

## Materials and Methods

### Patients and specimens

A total of 133 breast cancer patients (aged 26–84 years, mean age 53 years) were recruited for this study from July to September 2008 in Tianjin Medical University Cancer Institute and Hospital (TMUCIH; Tianjin, China). Among them, 116 (87.2%) patients underwent unilateral radical mastectomy and dissection of axillary lymph nodes, 10 (7.5%) patients underwent local tumorectomy or breast-conserving surgery, and 7 (5.3%) patients did not undergo surgery. All diagnoses were confirmed based on pathological examination and 2003 WHO Classification of Tumors of the Breast, and 125 (94.0%) cases were invasive ductal carcinoma and 8 (6.0%) were ductal carcinoma in situ. Detailed clinicopathological information including menopausal status, clinical stage, tumor size, histological grade, lymph node involvement, as well as estrogen receptor (ER) and progesterone receptor (PR) status is presented in Table 3. ER and PR status were determined by immunohistochemical staining and defined as positive if more than 15% of tumor cells showed positive nuclear staining.

**Table 3:** Clinicopathological characteristics of breast cancer patients (N = 133).

Characteristics	Cases (%)
Menopausal status	
Premenopausal	60 (45.1)
Postmenopausal	73 (54.9)
Pathology diagnosis	
Invasive ductal carcinoma	125 (94.0)
ductal carcinoma in situ	8 (6.0)
Clinical Stage	
I	40 (30.1)
II	80 (60.1)
III	4 (3.0)
Missing	9 (6.8)
Tumor size (cm)	
≤2	44 (33.1)
2–5	68 (51.1)
>5	3 (2.3)
Missing	18 (13.5)
Lymph nodes involvement	
Negative	75 (56.4)
Positive	48 (36.1)
Missing	10 (7.5)
Histology grade	
I	9 (6.8)
II	77 (57.9)
III	14 (10.5)
Missing	33 (24.8)
ER status	
Positive	71 (53.4)
Negative	53 (39.8)
Missing	9 (6.8)
PR status	
Positive	40 (30.0)
Negative	84 (63.2)
Missing	9 (6.8)

Note: “Missing” indicates the number (%) of cases for which the corresponding information was not available.

From each individual, 2 ml fasting peripheral blood was collected into BD Vacutainer tubes without anticoagulant (BD Biosciences, Franklin Lakes, NJ, USA) before surgery. The blood was centrifuged at 3,000 g for 10 min at room temperature, and serum was then stocked in 0.5 ml aliquots and stored at −80°C. The study protocol was approved by the Institutional Review Board of Tianjin Medical University Cancer Institute and Hospital (TMUCIH; Tianjin, China) and written consent was obtained from all participants.

### Cell culture

MDA-MB-435 cell line was obtained from the American Type Culture Collection (ATCC). *ERBB2*-transfected MDA-MB-435 (435/*ERBB2*) cell line was a gift from Dr. Mien-Chie Hung and Dr. Wei Zhang (University of Texas M. D. Anderson Cancer Center, Houston, TX). The cells were cultured in DMEM-F12 Medium (GIBCO, Carlsbad, CA, USA) supplemented with 10% newborn calf serum (GIBCO), 100 U/ml penicillin and 100 µg/ml streptomycin. The cells grown to 80% confluence were used for experiments.

### Western blot assay

Cancer tissues or cultured cells were lysed with protein lysis buffer containing 20 mM Tris-HCl pH 7.4, 5 mM EDTA, 1% Triton-X 100, 150 mM NaCl, 1% DTT, and 1% protease inhibitor cocktail (Sigma, St. Louis, MO, USA). The proteins in the lysate were separated by SDS-PAGE and transferred to polyvinyldifluoride membranes (Pierce, Rockford, IL, USA). The membranes were blocked in 5% skimmed milk in TBST (10 mM Tris, 150 mM NaCl, 0.05% Tween 20, pH 8.3) for 1 hour at room temperature, then incubated with a 1∶1000 dilution of rabbit polyclonal HER2 antibody (A0485, DAKO, Denmark) in 1% skimmed milk in TBST at 4°C overnight. The following day, the membranes were washed in TBST and then incubated with HRP-conjugated goat anti-rabbit (GE Healthcare, Piscataway, NJ, USA) at a dilution of 1∶2500 for 45 min at room temperature. The membranes were then washed in TBST and immunoreactive protein bands were visualized by enhanced chemiluminescence (ECL) reagents (GE Healthcare).

### Immunohistochemistry

Breast cancer tissue was obtained by either breast core-needle biopsy or surgery. Immunohistochemistry (IHC) staining of specimens was carried out on formalin-fixed paraffin-embedded tissues using the polyclonal rabbit HER2 antibody (A0485; DAKO) at a dilution of 1∶400, and a peroxidase-conjugated detection system (DAKO). Development was performed with diaminobenzidine, using hematoxylin counterstaining. HER2 IHC staining was scored as (0), weak positive (1+), moderate positive (2+) and strong positive (3+) based on the percentage of cells stained as positive and staining intensity following the standard of DAKO Hercept Test™ (Fig. 1).

### Dot blot assay

Two µl serum was diluted 100-fold with TBS buffer and then spotted onto nitrocellulose membrane with Bio-Dot Microfiltration Apparatus (Bio-Red, Hercules, CA, USA). All samples were deposited in random order on the 48-spot membrane. In addition, HER2 from MDA-MB-435/*ERBB2* cell lysate with a known concentration was diluted as 1∶5^2^, 1∶5^3^, 1∶5^4^, 1∶5^5^, 1∶5^6^, and 1∶5^7^ (0.2, 1, 5, 25, 125 and 675 ng/ml) to be used as extrapolation standards. The membranes were probed with the same antibody as for western blot. The light intensity of a single spot on the membrane was detected using a ChemiDocXRS imaging system (Bio-Rad). Based on the light intensities of HER2 protein concentration standards, the concentration of serum HER2 in each spot was calculated. All samples were performed in duplicates (Fig. 2).

### Enzyme-linked immunosorbent assay

In cases where serum HER2 level, detected by dot blot, was consistent with tissue HER2 status examined by IHC, the HER2 level was also measured using a sandwich ELISA Kit (Bender MedSystem, Vienna, Austria) following manufacturer's instructions. 100 µl of diluted serum samples, controls, and standards were micropipetted in the designated wells, and then 50 µl of HRP-Conjugate were added into each well and incubated at room temperature for 2 h. After washing, the substrate tetramethylbenzidine (TMB) was added into the wells and the reaction was terminated by addition of Stop Solution, and absorbance was measured at 450 nm. All experiments were performed in a blinded manner and in duplicates, and the HER2 concentration was calculated using their average optical densities based on standard curves.

### Statistical analyses

All statistical analyses were performed with Statistical Package for the Social Sciences (SPSS, version 13.0). Spearman rank correlation and rank sum test was used to analyze the correlations between HER2 levels detected by IHC, dot blot or ELISA. Chi-square (χ^2^) test or Fisher's exact test, and rank sum test was used to compare the serum HER2 level among cancer patients with various clinicopathological parameters. *P*-values of less than 0.05 were considered as statistically significant.
